# Long-term effects of low-intensity training with slow movement on motor function of elderly patients: a prospective observational study

**DOI:** 10.1186/s12199-019-0798-4

**Published:** 2019-06-13

**Authors:** Kanae Kanda, Yutaka Mori, Kunihisa Yamasaki, Hiroko Kitano, Aya Kanda, Tomohiro Hirao

**Affiliations:** 1 Department of Public Health, Faculty of Medicine Kagawa University, 1750-1 Ikenobe Miki-cho Kita-gun, Takamatsu, Kagawa 761-0793 Japan; 2 Sin Cire Co., Ltd, 14-29 Ogi-machi, Daito, Osaka, 574-0033 Japan

**Keywords:** Body weight, Frail elderly, Long term, Motor function, Slow movement, Training

## Abstract

**Background:**

Slow-motion training, which comprises exercising using extremely slow-movements, yields a training effect like that of high-intensity training, even when the applied load is small. We developed a slow-training exercise program that allows elderly people to safely use their own body weight without a machine. Previously, it was confirmed that functional gait and lower limb muscle strength were improved by low-intensity training using bodyweight training for 3 months. This study evaluated the long-term effects of low-intensity training using body weight with slow-movements on the motor function of frail, elderly patients.

**Methods:**

Ninety-six elderly men and women aged 65 years or older whose level of nursing care was classified as either support required (1 and 2) or long-term care required (care levels 1 and 2) volunteered to participate. Two facilities were used. Participants at the first facility used low-intensity training using body weight with slow-movements (low-stress training [LST] group, *n* = 65), and participants at another facility used machine training (MT group, *n* = 31). Exercise interventions were conducted for 12 months, once or twice per week, depending on the required level of nursing care. Changes in motor function were examined.

**Results:**

Post-intervention measurements based on the results of the chair-stand test after 12 months showed significant improvements from pre-intervention levels (*P* < 0.0001) in the LST group and MT group. Although the ability of performing the Timed Up & Go test and the ability to stand on one leg with eyes open improved in both groups, no significant change was observed. When changes after 12 months were compared between the two groups, no significant difference was observed for any variables.

**Conclusions:**

Slow body weight training for 12 months without a machine improved the lower limb muscle strength. Therefore, it could have the same effects as training using a machine.

**Trial registration:**

UMIN000030853. Registered 17 January 2018 (retrospectively registered).

## Background

Declining motor function due to aging increases nursing care needs and the risk of falls, and it has various effects on health, life expectancy, and life prognoses. Muscle strength in the lower limbs alters faster than in the upper limbs, and it begins to decline rapidly around 60 years of age [1]. Meta-analyses from previous studies suggests that muscular strength training for the elderly improves body function, even in the elderly population with significant functional decline, thus establishing the possibility of improving muscle function with strength training [2, 3]. Recently, low-intensity exercise with slow-movement and tonic force generation (low-stress training [LST]) [4], which involves slow-movements while maintaining muscle tension that allows the achievement of substantial muscle hypertrophy using a relatively light load, has been developed, and evidence of its effects are being continuously accumulated. LST is a collective term that refers to muscle training during which the load is increased and decreased extremely slowly. We developed a slow-motion weight training program using one’s own body weight that can be easily performed by frail elderly subjects at home. In a previous study, it was confirmed that walking function and lower limb muscle strength were improved by slow training using one’s own body weight for 3 months [5]. In this study, exercise training was performed by elderly individuals for 12 months, and whether long-term improvements were achieved was examined.

## Methods

### Participants

**Fig. 1:**
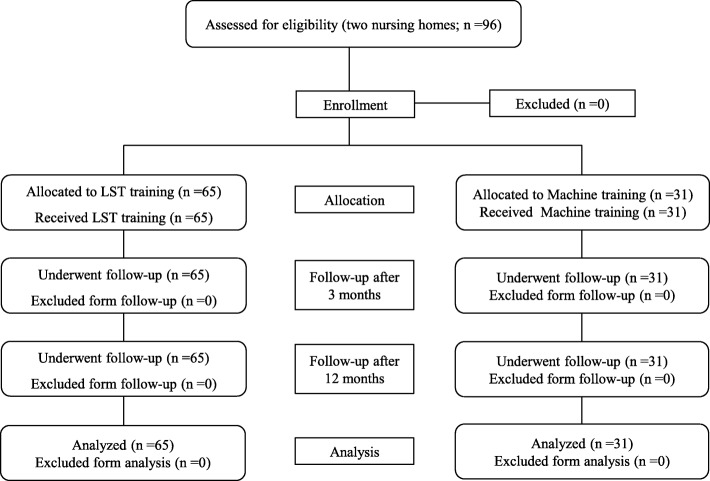
Flowchart detailing the timeline for participants

This study included elderly men and women aged 65 years or older, living at nursing care facilities in Osaka, who required a level of nursing care classified as either support required (care levels 1 and 2) or long-term care required (care levels 1 and 2). Participants who met any of the following criteria were excluded from the study: (1) those who had difficulty participating in the exercise program because of apparent cognitive symptoms, (2) those who had physical limitations due to effects of disease aggravation or when discontinuation of the ongoing program was determined to be a better option, (3) when it became difficult for the participant to continue the exercise program on a regular basis; (4) when the attending physician determined that the participant had to stop using day services because of the effects of diseases, and (5) other people who the physician in charge of the research program determined as being inappropriate for inclusion. The participants were recruited between March and August 2016 from two facilities. Participants at the first facility performed exercise rehabilitation therapy using LST (the LST group), and participants at the other facility performed exercise rehabilitation therapy using a machine (the control group). The study flowchart is shown in Fig. 1. Exercise programs and exercise machines were provided by Sin Cire Co., Ltd.

**Fig. 2:**
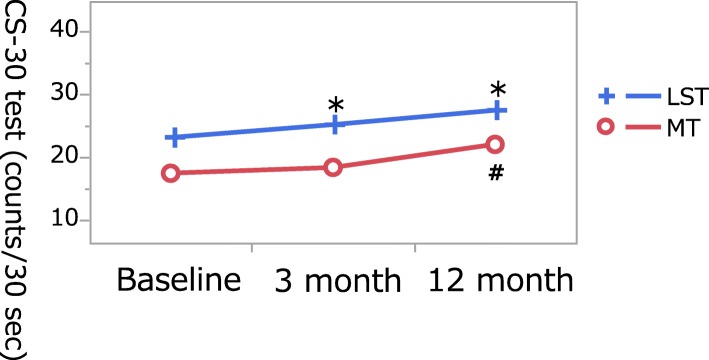
Change in the chair-stand test (the CS-30 test) score following LST training. Two-way repeated measures were used to compare values between the two groups in intra-individual variation. Data are presented as mean (95% confidence interval). LST(*), MT (#); *P* < 0.05. *P* values less than 5% were considered significant in both cases. LST low-stress training (low-intensity exercise with slow movement and tonic force generation), MT machine training, SD standard deviation

### Interventions (LST group)

We developed an LST program based on a previous program [5]; for 12 months, participants underwent the exercise interventions once or twice per week, depending on the individual’s required level of nursing care. Regarding the amount of physical exercise, two sets of six different types of exercise (to strengthen the thighs, lower legs, buttocks, abdomen, chest, and back) were performed at a pace of eight times per minute (all exercises were performed with a hold time of 1–2 s with a rest period of 1–2 s); the sets of exercises were separated by 1-min breaks. Specifically, the training consisted of the following: thigh exercises involved participants holding a bar with both hands while in the standing position and performing squats while sitting on a chair, lower leg exercises involved participants holding a bar with both hands while in the standing position and performing a calf raise, gluteal exercises involved participants holding a bar with one hand while in the standing position and performing a knee-up by using one leg each time, and abdominal exercises involved participants placing a ball in front of their abdomen while in the sitting position, tilting the upper half of their body forward while holding the ball with their hands, and performing abdominal muscle exercises. Additionally, chest exercises involved participants sandwiching a ball between the back and the back of a chair, raising the arms to the height of the chest, pulling the elbows backwards against the scapulae, and sticking out the chest. Back exercises involved participants squeezing a ball with the back while sticking out the chest and sandwiching the ball between the back and the back of the chair. All exercises were performed by combining slow-movements and 3-s intervals of rhythmic breathing. Each participant was instructed to consciously maintain muscle activity as long as possible. All participants were instructed to follow the same exercise program under the guidance of a specialized instructor.

### Control (machine training group)

The control group was subjected to low-intensity machine training (MT) using a normal speed. The MT consisted of a program combining body area-specific muscle strength-training exercises using a hydraulic weight-training machine [6–8]. Regarding the amount of physical exercise, two sets of seven different types of exercise (to strengthen the thighs, lower legs, buttocks, chest, back, shoulders, and abdomen) were performed at a pace of 15 times per minute (all exercises were performed with a hold time of 1–2 s with a rest period of 1–2 s), and the sets were separated by 1-min breaks. Details of the muscle training were as follows: (1) thigh (femoral region) exercise involved flexion and extension movements of the knee joint performed using a Leg Extension/Curl® machine (GH-104; ALPS Electric Co., Ltd., Tokyo, Japan), (2) lower leg exercise involved up-and-down movements of the lower extremities performed using a Stepster® (TB-699; Takada-Bed Co., Ltd., Sennan, Japan), (3) buttocks (gluteal region) exercise involved abduction and adduction movements of the hip joint performed using an Abduction/Adduction® (GH-102; ALPS Electric Co., Ltd., Tokyo, Japan). (4) chest (thoracic region) exercise comprised a chest press performed using a home gym machine, (5) back (dorsal region) exercise comprised a pull-down performed using a home trainer, (6) shoulder exercise involved arm raising and lowering movements performed using a home trainer, and (7) arm exercise involved arm curls performed using a home trainer. A single training device (YMHT-250; Yamato Human Co. Ltd., Tokyo, Japan) was used for the latter four exercises.

### Measurements

The primary outcome was the difference in mean values obtained from the Timed Up & Go test (TUG) used to evaluate the ability to perform compound motions. To determine TUG results [9, 10], we measured the time required to rise from the chair and stand, walk to the landmark 3 m ahead, and return to the chair and sit again. During each session, the measurements were performed twice, and the best value was recorded.

The secondary outcomes were the differences in mean values of the chair-stand test (lower-limb muscle strength) and of the one-leg standing test with eyes open (balance ability). The chair-stand test comprised the CS-30 test [11], during which the number of times the participant sat down and stood up within a 30-s period was measured once. For the one-leg standing test with eyes open [12], we measured the time during which the participant was able to lift the foot from the floor and maintain it in the same position. A duration of 60 s was considered the maximum, and each measurement was performed twice.

Participant characteristics that were recorded included their age at study initiation, sex, level of nursing care required, body weight, and blood pressure at rest. The body weight of each participant was measured using a Tanita body composition meter (BC-705N; Tanita Corp., Tokyo, Japan). The blood pressure of each participant at rest (systolic pressure and diastolic pressure) was measured in the sitting position using a Terumo electronic blood pressure monitor (ES-P2000; Terumo Corp., Tokyo, Japan) after the participant had rested for 5 min.

### Sample size calculation

To observe significance levels (alpha = 0.05, power = 0.8, standard deviation = 5.0, effect size = 3.0) that reflected the average differences in the TUG, which was the primary outcome, it was estimated that each group required 45 participants [13–15]; this sample size was calculated using JMP Pro 12.0 (SAS Institute Inc., Cary, NC, USA).

### Statistical analysis

The results are expressed as the mean value ± standard deviation, minimum value, and maximum value. Comparisons between values measured before the intervention and those measured after 3 months and 12 months were performed using the repeated measures analysis of variance. Comparisons between the two groups in terms of variations were performed using an unpaired *t* test. Hazard ratios less than 5% were considered significant for both groups. The statistical analysis was performed using JMP Pro 13.0.

### Ethics statement

Informed consent was obtained from the participants. This study was approved by the Ethics Committee of Kagawa University Faculty of Medicine and Graduate School of Medicine (approval number: Heisei28–128).

## Results

The baseline characteristics of the study participants are shown in Table 1. There were 65 participants in the LST group (mean age 80.6 ± 6.1 years) and 31 in the control group (mean age 80.4 ± 5.7 years). To assess the current medical history, information regarding major diseases, for which nursing care was required, was collected. Among these diseases, joint diseases were the most common, followed by hypertensive diseases. All participants completed the 12-month exercise training program. Before the intervention, the values of each index showed no significant differences between the two groups. The CS-30 test results were only significantly different between the two groups at baseline. For both groups, the average number of weekly exercise sessions performed by the study participants were 1.8 times per week. No adverse events resulting from participation in the exercise training programs were reported.

**Table 1:** Baseline characteristics of the study participants

Baseline variables	LST group (*n* = 65)			MT group (*n* = 31)			*P* value
	Mean ± SD	Minimum	Maximum	Mean ± SD	Minimum	Maximum	
Female sex (%)	50 (76.9)			25 (80.6)			0.6838
Age (years)	80.6 ± 6.1	66	93	80.4 ± 5.7	70	91	0.8809
Body weight (kg)	54.7 ± 10.3	30.4	84.3	57.3 ± 11.3	36.4	80.1	0.2799
Systolic blood pressure (mmHg)	131.2 ± 18.1	95	185	129.9 ± 21.	95	181	0.7540
Diastolic blood pressure (mmHg)	67.6 ± 10.7	41	92	68.6 ± 10.7	43	89	0.6710
Timed Up & Go test (s)	12.2 ± 5.7	6.4	32.0	14.7 ± 6.4	6.5	31.8	0.0567
One-leg balance with an open eye (s)	11.4 ± 13.0	0.37	60.0	16.5 ± 21.6	1.02	60.0	0.1534
CS-30 test (counts/30 s)	23.1 ± 7.8	2	43	17.4 ± 7.3	6	32	*0.0011*
Smoking status (%)	1 (1.5)			2 (6.4)			0.1997
Current medical history (multiple answers)							
Cerebrovascular diseases	16			9			
Heart diseases	13			7			
Malignant neoplasm	9			6			
Respiratory diseases	5			2			
Joint diseases	61			22			
Dementia	5			2			
Parkinson disease	2			0			
Diabetes	7			3			
Visual/hearing disorder	17			3			
Fracture/fall	15			8			
Spinal cord injury	1			0			
Hypertensive diseases	22			12			
Others	26			14			

Values are presented as means ± standard deviation, minimum value, and maximum value

All *P* values compared the characteristics of the LST and MT groups using unpaired *t* tests or Chi-squared tests

*LST* low-stress training (low-intensity exercise with slow movement and tonic force generation), *MT* machine training, *SD* standard deviation

Participants’ motor functions prior to the LST program were compared using repeated measures with that after 3 months and 12 months of training; the results showed significant improvements in the chair-stand test (comprised the CS-30 test) performance (23.1 ± 7.8 times before the exercise training program and 25.1 ± 7.8 times at 3 months, and 27.4 ± 9.2 times at 12 months; *P* < 0.0001) (Table 2, Fig. 2). The TUG results indicated no significant changes in the ability to perform compound motions (mean, 12.2 ± 5.7 s prior to the exercise training program and 11.6 ± 5.7 times at 3 months, and 12.2 ± 7.8 s at 12 months; *P* = 0.3351). Although the ability to stand on one leg with eyes open tended to improve, no significant change was found (pre-intervention mean 11.4 ± 13.1 s; mean at 3 months 12.8 ± 14.2 s, mean at 12-months 13.4 ± 15.1 s; *P* = 0.2124). A comparison of blood pressure at rest using the values prior to the exercise training program showed that body weight, systolic pressure, and diastolic blood pressure changed significantly for the LST group after 12 months of exercise.

**Table 2:** Comparisons between values measured before intervention and those measured 3 months later and 12 months later

	Baseline Mean ± SD	3 months Mean ± SD	12 months Mean ± SD	*P* value
LST group (*n* = 65)				
Body weight (kg)	54.7 ± 10.4	54.4 ± 10.6	55.2 ± 11.1	*0.0225*
Systolic blood pressure (mmHg)	131.2 ± 18.2	122.7 ± 17.5	125.8 ± 14.3	*0.0008*
Diastolic blood pressure (mmHg)	67.6 ± 10.7	63.9 ± 10.8	66.7 ± 10.7	*0.0236*
Timed Up & Go test (s)	12.2 ± 5.7	11.6 ± 5.6	12.2 ± 7.8	0.3351
One leg balance with an open eye (s)	11.4 ± 13.1	12.8 ± 14.2	13.4 ± 15.2	0.2124
CS-30 test (counts/30 s)	23.1 ± 7.8	25.1 ± 7.8	27.4 ± 9.2	*< .0001*
MT group (*n* = 31)				
Body weight (kg)	57.3 ± 11.3	57.8 ± 11.4	57.7 ± 11.1	0.6426
Systolic blood pressure (mmHg)	129.9 ± 21.0	130.0 ± 21.6	123.5 ± 18.0	*0.0273*
Diastolic blood pressure (mmHg)	68.6 ± 10.7	69.8 ± 12.4	66.3 ± 9.7	0.1211
Timed Up & Go test (s)	14.7 ± 6.4	13.7 ± 5.7	14.3 ± 9.5	0.5564
One leg balance with an open eye (s)	16.5 ± 21.6	15.0 ± 21.0	10.3 ± 15.2	0.2191
CS-30 test (counts/30 s)	17.4 ± 7.3	18.3 ± 7.6	22.0 ± 9.1	*< .0001*

Data are presented as the mean ± standard deviation and mean change

One-way repeated measures ANOVA were used to compare values measured before the intervention and those measured at 3 months and 12 months

*P* < 0.05. *P* < 5% was considered significant in both groups

*LST* low-stress training (low-intensity exercise with slow movement and tonic force generation), *MT* machine training

For the MT group, the results showed significant improvements in the chair-stand test results (17.4 ± 7.3 times prior to the exercise training program and 18.3 ± 7.6 times at 3 months, and 22.0 ± 9.1 times at 12 months; *P* < 0.0001). The TUG results indicated no significant changes (mean, 14.7 ± 6.4 s prior to the exercise training program and 13.7 ± 5.7 times at 3 months, and 14.3 ± 9.5 times at 12 months; *P* = 0.5564). Although the ability to stand on one leg with eyes open seemed to improve, no significant change was found (pre-intervention mean 16.5 ± 21.6 s; mean 15.0 ± 21.0 s at 3 months, 10.3 ± 15.2 s at 3 months; *P* = 0.2191). A comparison of blood pressure at rest using the values found prior to the exercise training program showed that systolic pressure had decreased significantly after 12 months of exercise. There was no significant change in body weight. However, significant improvements were observed in systolic blood pressure and the ability to rise from the chair as a result of training conducted for 12 months in both groups. Based on the pre-intervention values and findings after 12 months of exercise, there were no significant differences in changes observed between the two groups (Table 3).

**Table 3:** Comparison between the two groups in terms of variation

	LST group (*n* = 65) Mean (95% CI)	MT group (*n* = 31) Mean (95% CI)	*P* value
Baseline – Month 3 later			
Body weight (kg)	− 0.30 (− 0.64 to 0.04)	0.49 (− 0.01 to 0.98)	*0.0105*
Systolic blood pressure (mmHg)	− 8.52 (− 12.76 to − 4.29)	0.09 (− 6.04 to 6.23)	*0.0239*
Diastolic blood pressure (mmHg)	− 3.74 (− 6.24 to − 1.23)	1.22 (− 2.40 to 4.85)	*0.0278*
Timed Up & Go test (s)	− 0.60 (− 1.22 to 0.01)	− 0.94 (− 1.83 to − 0.04)	0.3311
One leg balance with an open eye (s)	1.42 (−1.51 to 4.34)	− 1.54 (− 5.77 to 2.69)	0.0910
CS-30 test (counts/30 s)	2.06 (0.91 to 3.22)	0.90 (− 0.77 to 2.58)	0.2613
Baseline – Month 12 later			
Body weight (kg)	− 0.51 (− 0.28 to 1.30)	0.41 (− 0.74 to 1.56)	0.8877
Systolic blood pressure (mmHg)	− 5.46 (− 9.61to − 1.32)	− 6.42 (− 12.42 to − 0.42)	0.7949
Diastolic blood pressure (mmHg)	− 0.89 (− 3.54 to 1.76)	− 2.32 (− 6.16 to 1.52)	0.5441
Timed Up & Go test (s)	0.07 (− 1.18 to 1.31)	− 0.39 (− 2.20 to 1.42)	0.2245
One leg balance with an open eye (s)	1.97 (− 1.90 to 5.83)	− 6.24 (− 11.84 to 0.64)	0.1294
CS-30 test (counts/30 s)	4.34 (2.84 to 5.84)	4.58 (2.41 to 6.75)	0.8559

An unpaired *t* test was used to compare values between the two groups in terms of variation

Data are presented as a mean (95% confidence interval)

*P* < 0.05. *P* < 5% was considered significant in both groups

*LST* low-stress training (low-intensity exercise with slow movement and tonic force generation), *MT* machine training

## Discussion

It was confirmed that muscle strength of the lower limbs improved with LST for 12 months. Previous studies have reported that LST using an exercise machine enlarges the quadriceps muscle of healthy elderly people [16]. As a mechanism to obtain muscle strengthening effects, repetitive slow exercise sustains muscle contraction constantly and limits muscle blood flow during LST. Oxygen saturation in the muscle is lowered and the secretion of growth hormones is increased [4]. Even with weight training, it was thought that a mechanism similar to LST improved leg muscle strength. The muscle strength of the lower limbs greatly affected the continuation of exercise [17]. For frail elderly people, whose task was to continue exercising, it was suggested that LST is useful for long-term maintenance and improvement of limb muscle strength.

Regarding the compound walking ability, although there was significant improvement at 3 months, it returned to the same level after 12 months as before training. Therefore, improvements in walking ability may not be sustainable in the long term. It is important for elderly individuals to try to maintain improvement in their walking ability because decreased walking speed is strongly related to decreased ability to perform activities of daily living [18]. Therefore, it was considered necessary to devise measures such as increasing variations in exercise programs and changing load settings according to the ability of the individual.

No significant change was observed in balance. Among the physical fitness factors, changes in balance conspicuously occur due to aging, and due to fluctuations in the center of gravity, individuals experience falls [19]. The LST program conducted during this study did not involve exercises to improve balance. To improve the balance of elderly individuals, it is necessary to consider the static and dynamic specificities of balance. Furthermore, it may be necessary to create static balance training programs that involve extending the holding time of balancing on one leg and decreasing the stair climbing time. Exercises associated with activities of daily living, including those involving backward and forward movements and changes in the center of gravity, might need to be added to exercise programs to improve balance in elderly individuals [20, 21]. In addition, it may be necessary to gradually increase the level of difficulty, rather than the quantity of exercises during training.

Individuals with significantly decreased resting blood pressure at 3 months after LST also had significantly decreased resting blood pressure at 12 months after LST. Some studies have also shown that the antihypertensive effects of physical exercise include certain notable mechanisms [22, 23] such as decreased circulating blood volume, decreased cardiac output, and increased hormone levels with antihypertensive effects. Our study suggested that performing low-intensity exercise on a regular basis may decrease blood pressure at rest. In addition, it also showed that compared with high-load exercise training, low-load slow-motion training is associated with greater suppression of increased blood pressure during exercise [24] and decreased the risk of musculoskeletal injury, suggesting that low-load slow-motion training can be performed relatively safely, even by elderly individuals and individuals with low physical strength.

As a result of training for 12 months, significant improvements in lower limb muscle strength and systolic blood pressure were observed in the LST group and in the MT group. No significant differences were found between the LST and MT group at the 3-month interval. Studies of active, healthy, elderly people compared weight training using body weight at normal speeds and slow speeds, and it was reported that upper limb muscle strength, lower extremity muscle strength, and maximum extension leg muscle strength improved in both groups [25]. In our study, elderly subjects performed training using chairs and bars. Our findings showed that such types of LST also improved motor function, and that the effects were similar to those of LST using exercise machines.

There were several limitations to this study. First, the measurements were limited to motor functions, and the study did not include any evaluation of cognitive functions or the risk of falls. Additionally, we could not measure the factors of diet that are important and related to frailty, and the number of remaining teeth [26]. We limited the measurement items to motor function in order to reduce burden on patients, but in future studies, it is necessary to scrutinize the measurement items related to frailty. Second, the exercised body parts were not same between the LST group and machine training group. Specifically, the machine training group performed shoulder and arm exercises in addition to lower extremity and trunk exercises, although the LST group performed lower extremity and trunk exercises intensively. There is a possibility of over or under evaluating the effect of machine training. Third, the underlying mechanism of the effects of LST is still unknown. As a main result of this study, we reported that exercise improves the leg muscle strength of elderly individuals. However, in order to measure leg muscle strength more accurately, it is necessary to measure muscle mass using ultrasonography, computed tomography, magnetic resonance imaging, and dual-energy x-ray absorptiometry, and it is desirable to clarify the mechanism of muscle strengthening [27]. To improve the motor function in elderly people, it is necessary to understand the characteristics of dysfunction and deterioration of elderly persons, and to further improve exercise programs.

## Conclusions

Improvements in lower limb muscle strength and blood pressure at rest were confirmed as short-term effects of LST for frail, elderly subjects. It was suggested that slow weight training using body weight could be performed safely by frail, elderly people. Long-term improvements as a result of exercise were observed.

## Data Availability

The datasets supporting the conclusions of this article are included within the article and its additional files.

## Abbreviations

LST: Low-stress training (low-intensity exercise with slow movement and tonic force generation)
MT: Machine training
TUG: Timed Up & Go
